# Leucine-rich alpha-2-glycoprotein-1, relevant with microvessel density, is an independent survival prognostic factor for stage III colorectal cancer patients: a retrospective analysis

**DOI:** 10.18632/oncotarget.16289

**Published:** 2017-03-16

**Authors:** De-Cong Sun, Yan Shi, Ling-Xiong Wang, Yao Lv, Quan-Li Han, Zhi-Kuan Wang, Guang-Hai Dai

**Affiliations:** ^1^ The Second Department of Oncology, Chinese PLA General Hospital, Beijing, China; ^2^ Current Adress: Cancer Center Key Laboratory, Chinese PLA General Hospital, Beijing, China

**Keywords:** leucine-rich alpha-2-glycoprotein-1, microvessel density, Stage III colorectal cancer, a retrospective analysis

## Abstract

**Background:**

Leucine-rich alpha-2-glycoprotein-1 (encoded by *LRG1*) has been shown to be involved in multiple cancer progression and angiogenesis. LRG1 has been shown to be one of the five plasma proteins that can be used for colorectal cancer (CRC) diagnosis. The objective of the current study was to explore relationship between LRG1 protein expression and microvessel density (MVD) in stage III CRC.

**Methods:**

A single-center retrospective analysis of all stage III CRC who underwent surgery and adjuvant chemotherapy was carried out. LRG1 and CD34 were tested in tumor tissues by immunohistochemistry (IHC).

**Results:**

LRG1 protein expression was significantly associated with MVD (*P* <0.001) and other clinicopathological parameters, including T stage (*P*=0.028), differentiation (*P*=0.035) and vascular invasion (*P*=0.007). Cox multivariate regression analysis showed that LRG1 protein expression was an independent poor predictive factor for both disease-free and overall survival.

**Conclusion:**

LRG1 protein expression can be used as a prognostic marker for stage III CRC along with its use as a diagnostic marker for CRC in general.

## INTRODUCTION

Colorectal cancer (CRC) is one of the most common malignancies and the leading causes of cancer related death in the world. It is estimated that in 2014, there were more than 136,000 cases newly diagnosed with colorectal cancer and more than fifty thousand CRC related deaths in the United States [1]. CRC are divided into stage I-IV according to the American Joint Committee on Cancer (AJCC) TNM stage system [2]. Stage III CRC are those cases with any T stage, N1-3 and no metastasis [2]. Stage III CRC can also be further divided into three more separate categories: IIIA, IIIB and IIIC [2]. The 5-year overall survival (OS) for stage IIIA-C CRC are about 85%, 60% and less than 40%, respectively [2].

Although huge progress has been made in the clinical treatment of stage III CRC, patients still suffer from poor prognosis because of postsurgical recurrence and metastases. It has been suggested that many factors are attributed to the increasing risk of recurrence and death, inclusive of poor differentiation, large tumor size, late TNM stage, somatic mutation in *BRAF* mutation [3]. The molecular mechanisms of CRC development and progression remain poorly understood. It is hence imperative to continue to elucidate risk factors and biomarkers for prognostic prediction in patient with CRC.

Leucine-rich-α2-glycoprotein 1 (LRG1) was first isolated from human serum as a trace protein in 1977 [4] and named it as leucine-rich 3.1-S-alpha2-glycoprotein. Analysis of the amino acid sequence revealed that LRG1 is composed of 312 amino acids [5], 66 of which are leucine. The 312 amino acid sequence can be divided into 13 segments, 8 of which exhibit a periodic distribution of leucine. The very high content of leucine (66 of 312 amino acids) is an unusual characteristic of this protein. Proteins that contain such repeated leucine pattern are called leucine-rich repeats (LRRs) family, and there are over 70 proteins in LRR family [6]. LRG1, a membrane-associated LRR family protein, has been predicted as a regular in many physiological and pathological processes, such as glucan synthesis, neutrophilic granulocytic differentiation, cell adhesion [7].

In recent years, LRG1 were revealed to play critical roles in cell survival, migration, invasion, and apoptosis [8, 9]. Studies have demonstrated that overexpression of LRG1 is associated with several types of tumors [10-12], and could be regarded as a diagnostic marker of cancers [13, 14]. Choi et al, performed proteomic analysis of plasma proteins of CRC and adenomatous polyps, revealing that LRG1 upregulated in plasma of CRC patients [15]. A new study showed that LRG1 express higher in CRC tissues than in normal tissues [16], and was related to epithelial to mesenchymal transition (EMT) and angiogenesis *via* by HIF-1α and VEGF. A study revealed that LRG1 promoted the pathogenic angiogenesis of retinal microvessels by modulating TGF-β pathway [17].

Although LRG1 has been implicated in cancer progression, the prognostic value in CRC and the relationship between LRG1 and microvessel destiny (MVD) remain unclear. In this study, we detected the expression of LRG1 and MVD in 312 stage III CRC tissues and matched non-cancerous tissues by immunohistochemistry, analyzed the correlation between clinicopathological parameters and disease-free survival (DFS) and overall survival (OS), assessed the prognostic efficacy of LRG1 in stage III CRC patients, and tested the correlation between LRG1 and VEGF.

## PATIENTS AND METHODS

### Clinical samples and database

In this study, 312 stage III formalin-fixed paraffin-embedded CRC tissue samples and the matched non-cancerous tissues samples were obtained between January 1, 2005 and January 1, 2010 from Chinese PLA General Hospital, Beijing, China. The inclusion criteria were patients who underwent radical resection of primary tumor with curative intent for CRC and received adjuvant chemotherapy administration after surgery. Histopathological staging was confirmed by a consulting pathologist (initial) through examinations of retrieved specimens. Patients were excluded if they had a history of another malignancy or did not have informed consent, or complete clinicopathological and follow-up data available. The demographic variables included the gender, age, and pathological variables included histological grade, tumor depth of invasion, nodal status and other important feathers were collected in the stage III CRC database. Survival data were confirmed at hospital visit and by telephone. This study was approved by the Ethics Review Board in the Chinese PLA General Hospital, Beijing, China.

### Tissue microarray (TMA) construction

The TMA slides included 312 paired CRC and adjacent non-tumorous tissues. Pathologists stained tissue paraffin blocks of CRC samples with hematoxylin-eosin to confirm the diagnoses and marked three fixed points which displayed the most typical histological characteristics and one fixed point which displayed normal grands structure under a microscope. Using a tissue array instrument (Minicore Excilone, Minicore, Great Britain), we diverted 600 μm cores from each donor block into a recipient block microarray. Each recipient paraffin blocks contained 96 dots. Then the paraffin-embedded CRC microarray were sliced into 3-4 μm and mounted onto glass slides dealt with positive electric charge in order to avoid loss of tissues.

### Immunohistochemistry (IHC)

IHC was performed according to the protocol of anti-LRG1 antibody (Abcam, MA, USA) and anti-CD34 antibody (Abcam, MA, USA). Briefly, paraffin sections were first dewaxed and then hydrated. After microwave-based antigen retrieval with 10 mM citrate buffer (pH 6.0), endogenous peroxidase activity was blocked with incubation of the slides in 3% H_2_O_2_, and non-specific binding sites were blocked with 10% goat serum. After blocking, the slides were incubated with primary anti-LRG1 antibody (1:150 dilution) and anti-CD34 antibody (1:150 dilution) overnight in a moist chamber at 4°C. Slides were washed in PBS for three times, before being incubated with HRP-conjugated secondary antibody for 0.5 hour. Then the slides were stained with the DAKO Liquid 3,’3-diaminobenzidine tetrahydrochloride (DAB). Finally, the slides were counter stained with hematoxylin and observed under microscope. Negative control slides omitting the primary antibodies were included in all assays.

### Immunostaining evaluation

LRG1 expression was quantified based on the percentage of positive tumor cells (scored as: 0, less than 5% positively-stained cells; 1, 5-25%; 2, 26-50%; 3, > 51-70%; 4, > 71%) and the intensity of staining (scored as: 0, negative staining; 1, weak staining; 2, moderate staining; 3, strong staining). The overall staining score was calculated by multiplying the percentage score and the staining intensity score, which ranged between 1 and 12. The scores were independently decided by two experienced pathologists (YL and SW), who were blinded to patients’ results and other clinicopathological parameters, and their scores were averaged to get a final immunostaining score for each sample. Subsequently, patients were divided into high and low expression subgroups, and the mean immunostaining score was chosen as the cut-off value for each protein.

### Assessment of MVD

The area with the highest vascular density (hotspot) was selected using a 4x objective lens. Microvessels with positive immunostaining for CD34 were counted in three hotspots per section, using a 20x objective lens (0.785 mm^2^), by two experienced pathologists (YL and SW), who were blinded to patients’ results and other clinicopathological features. The vessel counts in each of the three hotspots were recorded as hotspot scores and then vessel counts per mm^2^ were calculated. After averaging the three vessel counts per mm^2^ for each hotspot, we obtained a final hotspot score for MVD.

### Statistical analysis

Statistical analyses were conducted using the SPSS 19.0 software (Chicago, IL, USA). Subjects were divided into the following categories: < 60 or ≥ 60 years of age; < 5.0 cm or ≥ 5.0 cm primary tumor; < 12 or ≥ 12 examined lymph nodes and high or low protein expression based on the individual cut-off score for each protein. The independent *t*-test was used to assess the association between LRG1 expression and MVD levels and also differences of LRG1 express levels between cancer tissues and corresponding non-cancerous tissues. Pearson’s χ2 test was used for examining the correlations between LRG1 expression level and the clinicopathological variables. For survival analyses, the Kaplan–Meier method was used to estimate the correlation between DFS, OS rates and clinicopathological variables. The log-rank test was used to compare survival curves. Univariate analyses were based on a Cox proportional hazard regression model. Multivariate analysis was used along with the Cox proportional hazard regression model to evaluate the independence of LRG1 in CRC prognosis and to calculate the hazard ratio (HR) with stepwise manner (forward: condition, entry α = 0.05, stay α = 0.1) and assess the odds ratio (OR). Differences were considered statistically significant if *P* value was less than 0.05.

## RESULTS

### LRG1 protein was robustly expression and was closely correlated with MVD in CRC tissues

**Figure 1:**
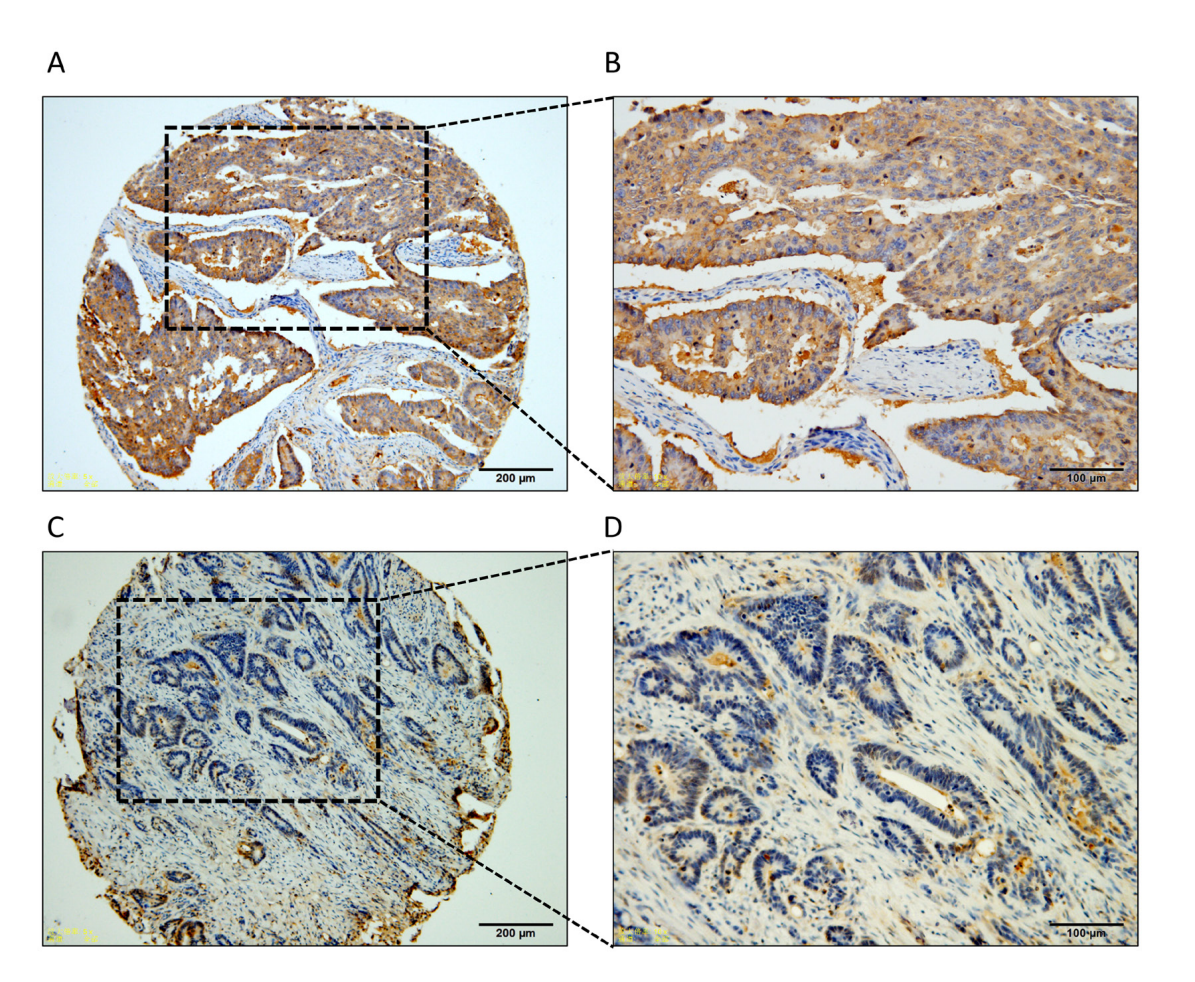
Representative IHC image of LRG1 expression in stage III CRC Low and high magnification images showing **A.**-**B.** high-level of LRG1 expression and **C.**-**D.** low-level of LRG1 expression.

To detect LRG1 expression and MVD in CRC tissues, 312 paraffin-embedded CRC samples were collected to construct TMA. As shown by the result of TMA-based IHC, immunoreactivities of LRG1 were mainly present in the cytoplasm in most of the cancer cells and occasionally observed in adjacent normal colorectal tissues. High expression levels of LRG1 were detected in 190 (60.9%) tumor specimens (Figure 1A, 1B ). In contrast, 122 (39.1%) tumor tissues expressed low levels of LRG1 (Figure 1C, 1D).

**Figure 2:**
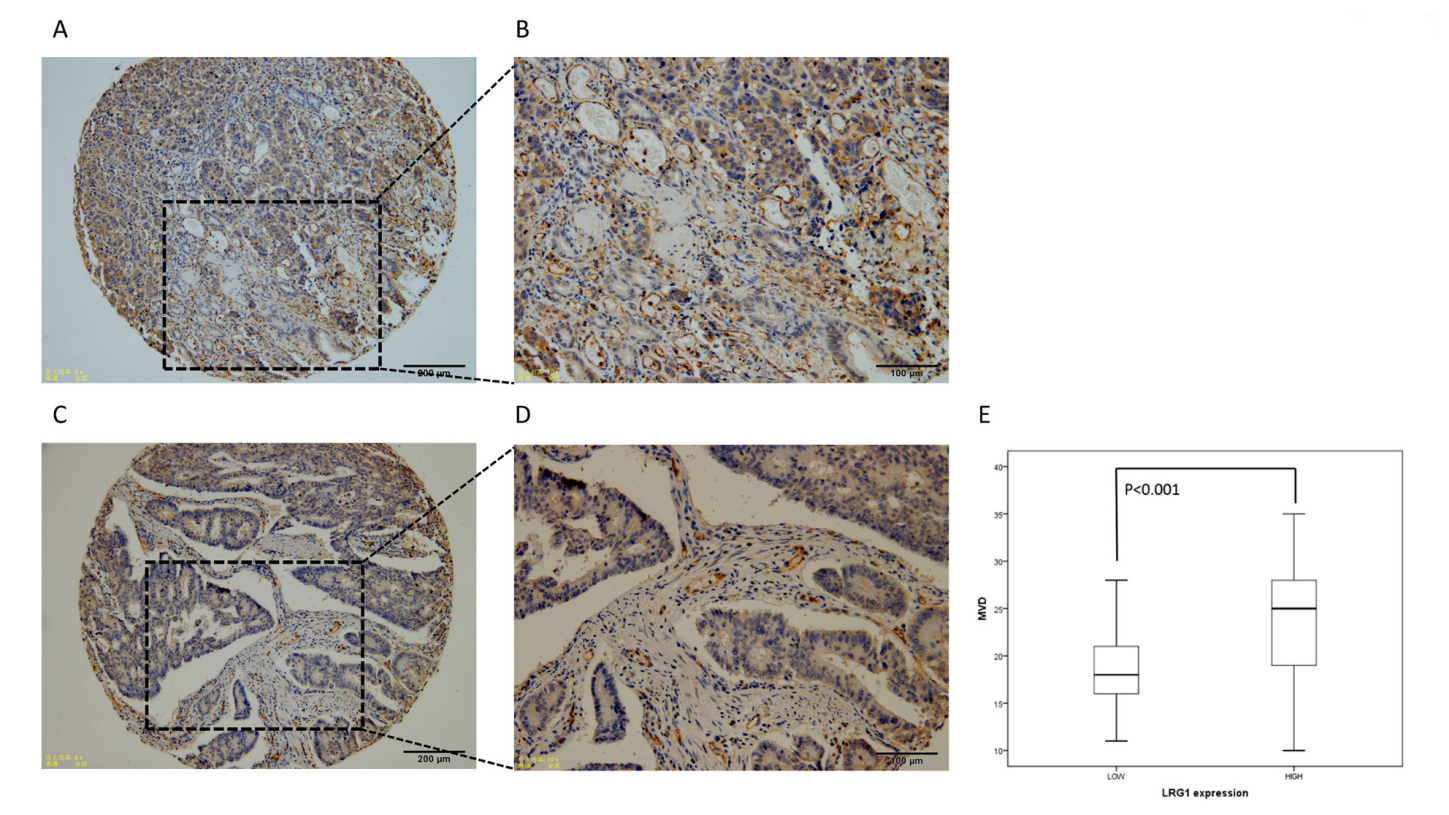
Representative IHC image of CD34 expression (MVD) in stage III CRC Low and high magnification images showing MVD ranged from 10 to 35/mm^2^ **A.**-**D. E.** Relationship between LRG1 expression and MVD.

**Figure 3:**
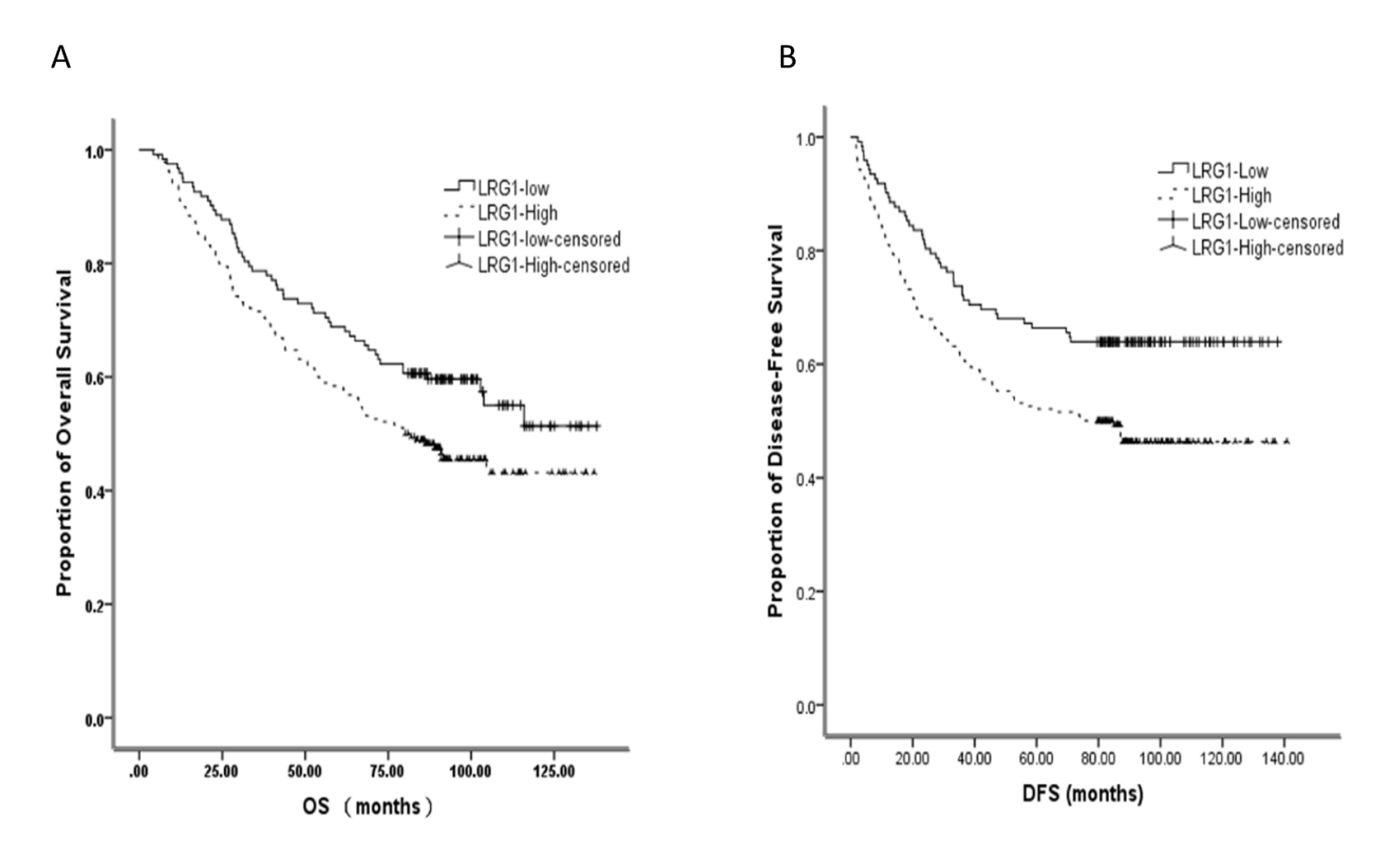
Kaplan–Meier plots showing correlation between expression of LRG1with overall and disease-free survival in the CRC patients

MVD were assessed by immunohistochemistry (Figure 2A-3D). MVD ranged from 10 (Figure 2C, 2D) to 35/mm^2^ (Figure 2A, 2B), the mean MVD was 21.66/mm^2^. The independent samples *t*-test was conducted to detect the correlation between two proteins expression. The result revealed that LRG1 expression was significantly associate with the MVD (*P* < 0.001) (Figure 2E and Table 1).

**Table 1:** Relationship between LRG1 expression and MVD.

LRG1 expression	N	MVD		
		Mean ± SD	t	*P*
LRG1-High	190	23.505±5.927	-8.603	<0.001
LRG1-Low	122	18.779±3.778		

Abbreviations: LRG1, Leucine-rich alpha-2-glycoprotein-1; MVD, microvessel density; SD, standard deviation.

### LRG1 expression level was significantly related to clinicopathological features

The clinicopathological variables of the 312 CRC patients were summarized in Table 2. Pearson’s χ2 test or Fisher’s exact test were performed to assess the association between clinicopathological features and the expression level of LRG1. The result explicated that the expression level was closely correlated with T stage (*P* = 0.028), differentiation (*P* = 0.035) and vascular invasion (*P* = 0.007).

**Table 2:** Relationship between LRG1 expression and clinicopathological features of 312 stage III CRC patients.

Variables	Cases	LRG1 expression		
	N=312	High	Low	*P*-value
Gender				0.655
Female	143	89	54	
Male	168	102	68	
Age (years)				0.065
<60	161	106	55	
≥60	151	84	67	
Tumor location				0.283
Right-sided	57	37	30	
Left-sided	245	153	92	
Differentiation				0.035*
Well/moderate	179	100	79	
Poor	132	90	43	
Surgical margin				0.605
Positive	18	12	6	
Negative	294	178	116	
Neural invasion				0.698
Positive	20	13	7	
Negative	292	177	115	
Vascular invasion				0.007*
Positive	50	39	11	
Negative	262	151	111	
Diameter of tumor				0.125
<5.0cm	184	119	65	
≥5.0cm	128	71	57	
T stage				0.028*
1-3	102	71	31	
4	210	119	91	
N stage				0.161
1	220	128	92	
2	92	62	30	
TNM stage				0.382
IIIA	30	19	11	
IIIB	190	110	80	
IIIC	92	61	31	

Abbreviations: LRG1, Leucine-rich alpha-2-glycoprotein-1.

### LRG1 is an independent prognostic factor for DFS and OS of stage III colorectal cancers

As it is demonstrated above, LRG1 expressed highly in stage III CRC tissues compared to adjacent colorectal mucosa and was clearly associated with differentiation, T stage, and vascular invasion, we next aimed to determine the correlation between LRG1 expression status and DFS and OS for CRC patients using the Kaplan–Meier method and compared the survival curves using the log-rank test. The OS of the high LRG1 expression group was significantly worse than that of the low group (Figure 3A). The same trend was also detected with DFS of 312 CRC patients (Figure 3B).

A univariate analysis revealed that the tumor location, pathological type, T/N/TNM stage, differentiation grade, vascular invasion, positive surgical margin, neural invasion were also predictors of DFS and/or OS in this study (Table 3). Furthermore, multivariate Cox proportional hazards regression analysis indicated that the age, TNM stage, differentiation grade, surgical margin, vascular invasion, tumor location and LRG1 expression level were independent prognostic factors of OS in this study (Table 3).

**Table 3:** Univariate analysis of factors associated with overall survival (OS) and disease-free survival (DFS) in 312 stage III CRC patients.

Variables	OS			DFS		
	Hazard	95% CI	*P*-value	Hazard	95% CI	*P*-value
Gender (male vs female)	1.639	1.188-2.260	0.003*	0.994	0.716-1.381	0.973
Age						
(for every 1 additional year)	1.024	1.010-1.036	0.000*	1.016	1.003-1.030	0.015*
Location (Right vs Left)	1.554	1.087-2.223	0.016*	1.477	1.016-2.148	0.041*
Differentiation						
(Poor vs Well/moderate)	1.811	1.318-2.488	0.000*	1.547	1.114-2.147	0.009*
Surgical margin	2.309	1.351-3.946	0.002*	1.956	1.104-3.463	0.021*
Neural invasion	2.104	1.252-3.535	0.005*	2.582	1.531-4.355	0.000*
Vascular invasion	1.551	1.029-2.338	0.036*	1.745	1.177-2.586	0.006*
Diameter of tumor	0.991	0.912-1.076	0.827	0.921	0.839-1.011	0.083
T stage (T4 vs T1-3)	2.123	1.449-3.110	0.000*	1.532	1.054-2.227	0.025*
N stage (N2 vs N1)	1.731	1.243-2.407	0.001*	1.435	1.018-2.022	0.039*
TNM stage IIIC vs IIIB vs IIIA	2.253	1.701-2.984	0.000*	1.732	1.303-2.303	0.000*
LRG1 expression (High vs Low)	1.441	1.031-2.014	0.032*	1.672	1.173-2.385	0.004*

Abbreviations: LRG1, Leucine-rich alpha-2-glycoprotein-1.

However, a multivariate analysis of the test cohort showed that age, TNM stage, tumor location, surgical margin, neural invasion, vascular invasion and also LRG1 expression level were independent prognostic factors of DFS for 312 colorectal cancers (Table 4). These results suggested that LRG1 was a prognostic factor for the OS of stage III CRCs.

**Table 4:** Multivariate analyses of factors associated with overall survival (OS) and disease-free survival (DFS) in 312 stage III CRC patients with the Cox proportional hazard regression model with stepwise manner (forward: condition, entry α=0.05, stay α=0.1).

Variables	OS			DFS		
	Hazard	95% CI	*P*-value	Hazard	95% CI	*P*-value
Gender (male vs female)	1.225	0.905-1.740	0.174	-	-	-
Age						
(for every 1 additional year)	1.013	1.000-1.027	0.047*	1.018	1.004-1.032	0.012*
Location (Right vs Left)	1.855	1.263-2.727	0.002*	1.692	1.134-2.524	0.010*
Differentiation						
(Poor vs Well/moderate)	1.211	1.018-1.761	0.036*	1.169	0.821-1.663	0.386
Surgical margin	1.964	1.171-2.598	0.040*	2.466	1.531-4.355	0.005*
Neural invasion	1.866	0.967-3.601	0.063	1.250	1.223-1.278	0.025*
Vascular invasion	2.415	1.694-4.366	0.026*	1.504	1.105-2.276	0.018*
T stage (T4 vs T1-3)	1.485	0.927-2.379	0.100	1.532	1.054-2.227	0.370
N stage (N2 vs N1)	1.169	0.844-1.542	0.741	1.435	1.018-2.022	0.554
TNM stage IIIC vs IIIB vs IIIA	2.037	1.222-3.396	0.006*	1.750	1.346-2.927	0.003*
LRG1 (High vs Low)	1.517	1.067-2.158	0.020*	1.754	1.410-2.53	0.013*

Abbreviations: LRG1, Leucine-rich alpha-2-glycoprotein-1.

## DISCUSSION

CRC is the second leading cause of cancer death in men and the third leading cause in women in the United States [18]. It is estimated that in 2014, there were more than 136,000 cases newly diagnosed with colorectal cancer and more than fifty thousand CRC related deaths in the United States [1]. CRC are divided into I-IV stage according to the American Joint Committee on Cancer (AJCC) TNM stage system [2]. Stage III CRC are those cases with any T stage, N1-3 and no metastasis [2]. The classical treatment for stage III CRC contains radical resection and postsurgical chemotherapy, which made stage III CRC less therapeutic heterogeneity than other stages. Thus, in this study we selected stage III CRC with completed clinicopathological feathers to study the relationship between LRG1 and prognosis.

Recently, studies revealed that LRG1 took part in multiple cancers progression and development. LRG1 expressed more highly in non-small cell lung cancer (NSCLC) [14], small cell lung cancer (SCLC) [19], hepatocellular carcinoma (HCC) (10), ovarian cancer [20], endometrial carcinoma [12] tissues and serum than matched normal spices. High LRG1 was also detected in NSCLC urinary exosomes [21]. LRG1 was significantly associated with cancer recurrence and poor prognosis, mainly because it played an important role in promoting cancer cell proliferation, invasion and migration through epithelial-to-mesenchymal transition (EMT) and Transforming growth factor-β (TGF-β) signaling pathway [8, 16].

Several studies have revealed the relationship between the prognosis and angiogenesis of CRC and LRG1. An elevated concentration of LRG1 in CRC was detected using proteomic analysis of plasma samples [22]. Proteomic biomarkers screening between adenomatous polyps and CRC patients revealed LRG1 is one of the upregulated serum biomarkers, which made LRG1 a novel biomarker for CRC development and tumorigenesis from colon adenoma [15]. Zhang et al demonstrated that LRG1 was overexpressed in CRC tissues and significantly associated with cancer T and N stage [16]. Furthermore, it was shown hypoxia-inducible factor-1 (HIF-1) induced LRG1-related angiogenesis. The mechanism of LRG1-related angiogenesis mainly focused on TGF-β signaling pathway [8]. By investigating choroidal neovascularization, researchers understood that *via* activation of TGF-β-ALK1 pathway, which successively activated Smad 1, 5 and 8, LRG1 promoted aberrant neovascularization [17].

In this study, we tested LRG1 and CD34 expression in CRC tissue *via* IHC, aiming at confirming association between LRG1 and MVD. The results demonstrated that LRG1 was significantly associated with MVD, which confirmed LRG1 play a significant role in CRC aberrant angiogenesis. We also analyzed the correlation between LRG1 expression level and clinicopathological variables and found that LRG1 was closely correlated with T stage, differentiation and vascular invasion. This result was almost the same with one study mentioned above, but we did not observe relevance between LRG1 and lymphatic metastasis [16]. By multivariate survival analysis, we found LRG1 expression level was an independent poor predictive factor for DFS and OS of stage III CRC. This result was a further validation of LRG1 performing an important role in CRC progression and angiogenesis.

As a retrospective analysis, our current study had some limitations; however, this is the first report of confirming the predictive value of LRG1 in DFS and OS. And initially, we found the correlation with LRG1 and MVD in stage III CRC, which developed the understanding of angiogenesis of LRG1. Further work, including prospective study and mechanism research should be performed to verify issues that were not explored in our study.

## Abbreviations

LRG1: Leucine-rich alpha-2-glycoprotein-1
CRC: colorectal cancer
MVD: microvessel density
IHC: immunohistochemistry
OS: overall survival
LRRs: leucine-rich repeats
EMT: epithelial to mesenchymal transition
TMA: Tissue microarray
